# Materials Cloud, a platform for open computational science

**DOI:** 10.1038/s41597-020-00637-5

**Published:** 2020-09-08

**Authors:** Leopold Talirz, Snehal Kumbhar, Elsa Passaro, Aliaksandr V. Yakutovich, Valeria Granata, Fernando Gargiulo, Marco Borelli, Martin Uhrin, Sebastiaan P. Huber, Spyros Zoupanos, Carl S. Adorf, Casper Welzel Andersen, Ole Schütt, Carlo A. Pignedoli, Daniele Passerone, Joost VandeVondele, Thomas C. Schulthess, Berend Smit, Giovanni Pizzi, Nicola Marzari

**Affiliations:** 1 National Centre for Computational Design and Discovery of Novel Materials (MARVEL), École Polytechnique Fédérale de Lausanne, CH-1015 Lausanne, Switzerland; 2 Theory and Simulation of Materials (THEOS), Faculté des Sciences et Techniques de l’Ingénieur, École Polytechnique Fédérale de Lausanne, CH-1015 Lausanne, Switzerland; 3 Laboratory of Molecular Simulation (LSMO), Institut des Sciences et Ingénierie Chimiques, Valais, École Polytechnique Fédérale de Lausanne, CH-1951 Sion, Switzerland; 4 nanotech@surfaces laboratory, Swiss Federal Laboratories for Materials Science and Technology (Empa), CH-8600 Dübendorf, Switzerland; 5 Swiss National Supercomputing Centre (CSCS), CH-6900 Lugano, Switzerland; 6 ETH, Zürich, Switzerland

**Keywords:** Materials science, Databases

## Abstract

Materials Cloud is a platform designed to enable open and seamless sharing of resources for computational science, driven by applications in materials modelling. It hosts (1) archival and dissemination services for raw and curated data, together with their provenance graph, (2) modelling services and virtual machines, (3) tools for data analytics, and pre-/post-processing, and (4) educational materials. Data is citable and archived persistently, providing a comprehensive embodiment of entire simulation pipelines (calculations performed, codes used, data generated) in the form of graphs that allow retracing and reproducing any computed result. When an AiiDA database is shared on Materials Cloud, peers can browse the interconnected record of simulations, download individual files or the full database, and start their research from the results of the original authors. The infrastructure is agnostic to the specific simulation codes used and can support diverse applications in computational science that transcend its initial materials domain.

## Introduction

Core to the mission of open computational science is the principle that open access to data, software and, eventually, infrastructure leads to scientific results that can be assessed, verified and reproduced. While the principle of reproducibility has long been at the foundation of science, information technology keeps pushing the limit of what is possible, and the challenge of translating this principle into sustainable practice is continuously evolving. Fortunately, funding agencies are increasingly aware of the need to develop comprehensive solutions to this challenge^1–5^, and guidelines for data management are being developed to help ensure that shared resources are easily findable, accessible, interoperable and re-usable (FAIR)^6^.

We believe this challenge calls for *open-science platforms* that let scientists use existing data and submit new content with minimal requirements on technical expertise. In this context, it is instructive to look at the field of software engineering, where platforms for sharing source code, such as GitHub (github.com), Bitbucket (bitbucket.org), or GitLab (gitlab.com) have already revolutionised the industry – not only in terms of the volume of source code that is shared publicly, but also in terms of how software developers interact and write code. These platforms are organised around Git, a software for “tracking changes in computer files and coordinating work on those files among multiple people” (en.wikipedia.org/wiki/Git). Besides hosting source code repositories, the platforms add a rich web interface for interactive browsing, controlling workflows, and collaboration through social interactions (sharing, commenting, mentioning, etc.). In our view, open-science platforms can learn from these successful examples, and have the potential to revolutionise the scientific discourse in similar ways. While these considerations apply to computational science in general, in the following we focus on the domain of materials.

The field of computational materials science is blessed in that research data is produced in digital form by default, and many of the necessary computational tools are available free of charge under open-source licenses. Over the last decade, substantial progress has been made in opening access to some of these resources: An early example is nanoHUB^7^, which provides access to interactive simulation tools as well as educational materials in the browser. Platforms have emerged that integrate data repositories with the software frameworks used to compute the data, such as AFLOWlib^8^ (with aflow), the Materials Project^9^ (with pymatgen, custodian, fireworks, atomate), OQMD^10^ (with qmpy), or JARVIS^11^ (with JARVIS-Tools). Finally, there are data repositories, such as NOMAD^12^, that collect and centralise large numbers of individual materials science calculations in one place.

However, the field still faces challenges in the context of open science. Materials simulations often rely on complex workflows which, e.g., combine simulations operating at different length- and time-scales and involve cycles of post-processing followed by further simulations. This calls for a flexible approach to designing such workflows, and to recording their many steps and interconnected results. Furthermore, screening a class of materials, even for one specific application, may involve running such workflows for thousands of candidate materials or more and require substantial computational power – the field of computational materials science is among the top consumers of high-performance computing resources around the world^13,14^. The substantial cost associated with executing such workflows makes efficient and complete record keeping highly valuable.

In our view, an open-science platform (OSP) should:

1. support and adopt open-source simulation codes and analytics tools;
2. provide an open-source framework for defining and managing computational workflows;
3. offer turnkey solutions based on open-source workflows and curated open datasets that are accessible to a diverse user base from computational science, experiments, and industrial R&D; and
4. enable FAIR sharing of data and workflows, facilitating reproducibility and encouraging extension and/or modification of published resources.

With this vision in mind, we have designed and implemented the Materials Cloud platform (materialscloud.org), which we describe in the remainder of this paper.

## Results

**Fig. 1:**
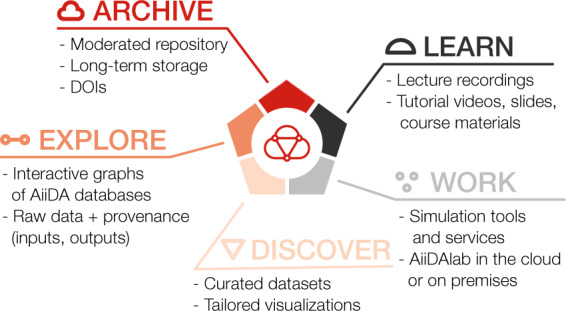
Materials Cloud organises its resources in five sections, LEARN, WORK, DISCOVER, EXPLORE, and ARCHIVE, representing different stages of the research life cycle.

Materials Cloud with its five sections – LEARN, WORK, DISCOVER, EXPLORE, and ARCHIVE – aims to provide an ecosystem that supports researchers throughout the life cycle of a scientific project, and helps them make their research output FAIR and reproducible. Figure 1 illustrates how the five sections of Materials Cloud mirror the typical research cycle, from learning to simulating and, finally, to curating and publishing results, which become the starting point for new research: LEARN (described in section Education and outreach) contains educational materials and videos; WORK (section Simulation services) focuses on simulation services, turnkey solutions and data analytics tools. The three sections DISCOVER, EXPLORE, and ARCHIVE are Materials Cloud’s approach to FAIR sharing of research data (sections FAIR data and Reproducibility).

Specifically, the ARCHIVE is a moderated repository, where researchers can submit relevant research data from computational materials science in formats of their choice. The repository guarantees long-term storage of records and associated metadata, their findability via persistent identifiers, and their accessibility via standard protocols. While the ARCHIVE is the port of entry for all research data hosted on Materials Cloud, it can form the basis for additional, interlinked layers of accessibility, interoperability and reusability: DISCOVER allows researchers to provide visualizations that are tailored specifically to their data and act as curated points of entry for discovering the contents of the data set, similar to figures in a paper. And for research done using the AiiDA workflow manager^15,16^, EXPLORE provides access to the raw, automatically recorded provenance graph through an interactive browser.

In the following, we present the individual sections of Materials Cloud in detail. For a brief overview, see Table 1.

**Table 1:** Detailed overview of Materials Cloud sections.

Section	Content	Target audience	Objective	Access	Submission
LEARN	Video lectures and tutorials	Students and experts in computational materials science	Dissemination of educational and research content	Open, no registration	Partners; other submissions considered
WORK	Online simulations tools and services; redeployable locally	Researchers in computational materials science	Dissemination of open simulation services and tools	Open, with registration for AiiDAlab	Partners; other submissions considered
DISCOVER	Curated datasets of calculated materials properties	Researchers in materials science	Dissemination of calculated materials properties	Open, no registration	Partners; other submissions considered
EXPLORE	AiiDA databases and their graphs	Researchers in computational materials science; data scientists	Exploration and query of the raw data and provenance of AiiDA workflows	Open, no registration	Open, with registration
ARCHIVE	Computational data in any format; experimental data linked to computational data	Researchers in materials science; data scientists	FAIR research data dissemination and storage	Open, no registration	Open, with registration

Submissions to ARCHIVE and EXPLORE are open to anyone, and moderated only for adherence to the scientific scope of the repository as well as basic data interoperability and reusability standards. Submissions to LEARN, WORK and DISCOVER are open to authors affiliated with Materials Cloud partners (materialscloud.org/home#partners) until the submission process is further streamlined, with other submissions being considered case by case.

### FAIR data: ARCHIVE and DISCOVER

The ARCHIVE and DISCOVER sections allow researchers to make their data available in a findable, accessible, interoperable, and reusable (FAIR) way^6^. The Materials Cloud ARCHIVE is an open-access, moderated repository for research data in computational materials science that allows researchers worldwide to upload and publish their data free of charge. In particular:

- it provides globally unique and persistent digital object identifiers (DOIs) for every record;
- metadata, such as title, description, keywords and license are always publicly available (Creative Commons Attribution Share-Alike 4.0 license);
- metadata can be harvested in a number of machine-readable formats, including HTML meta tags (Dublin core), OAI-PMH (Dublin core) and JSON-LD (schema.org);
- all data are stored at the Swiss National Supercomputing Centre (CSCS);
- it is non-commercial and free of charge;
- data records are guaranteed to be preserved for at least 10 years after deposition (already paid for);
- current size limits are 5 GB for general data records and 50 GB for AiiDA databases;
- moderators can approve larger data sets upon request (currently, 0.5 petabytes are allocated overall).

Data management plans (DMPs) that describe the handling of data both during a research project and after its completion are becoming standard components of research grant proposals. The Materials Cloud ARCHIVE is listed on the re3data^17^ and FAIR sharing^18^ repository registries, indexed by Google Dataset Search and B2FIND (b2find.eudat.eu), and it is a recommended repository for materials science by Nature Scientific Data. It complies with the data repository requirements of major funding agencies, and provides tailored DMP templates (materialscloud.org/dmp).

We take data stewardship seriously. In order to ensure data longevity, our storage provider CSCS is paid in advance to preserve any data set for at least 10 years after deposition, and we remain committed to continue data retention beyond this time period, if funding is available. For the unlikely scenario that a complete halt in funding for Materials Cloud prevents us from keeping the platform online, we have developed a contingency plan leveraging the long-term storage (LTS) system at CSCS. The LTS will guarantee that all registered DOIs continue to resolve to static landing pages describing the record metadata, with links to the data sets themselves.

Unlike *interdisciplinary* repositories for research data, such as Zenodo (zenodo.org), Data Dryad (datadryad.org), the Open Science Framework (osf.io), or figshare (figshare.com), the ARCHIVE is *moderated* and focuses on providing added value for datasets from computational materials science. Submissions to the ARCHIVE are expected to provide data that is of value to and can be used by other researchers in the field, such as data supporting a past, present or future peer-reviewed paper. Materials Cloud moderators are subject experts, who follow a set of criteria (materialscloud.org/moderation) to flag unsuitable or duplicate content, inappropriate form or topic, or excessive submission rates, much in the spirit of the arXiv preprint server (arxiv.org). While all data formats are accepted, moderators will suggest alternative formats, where applicable, that improve interoperability and reusability, in line with the 5-star deployment scheme to open web data (5stardata.info).

**Fig. 2:**
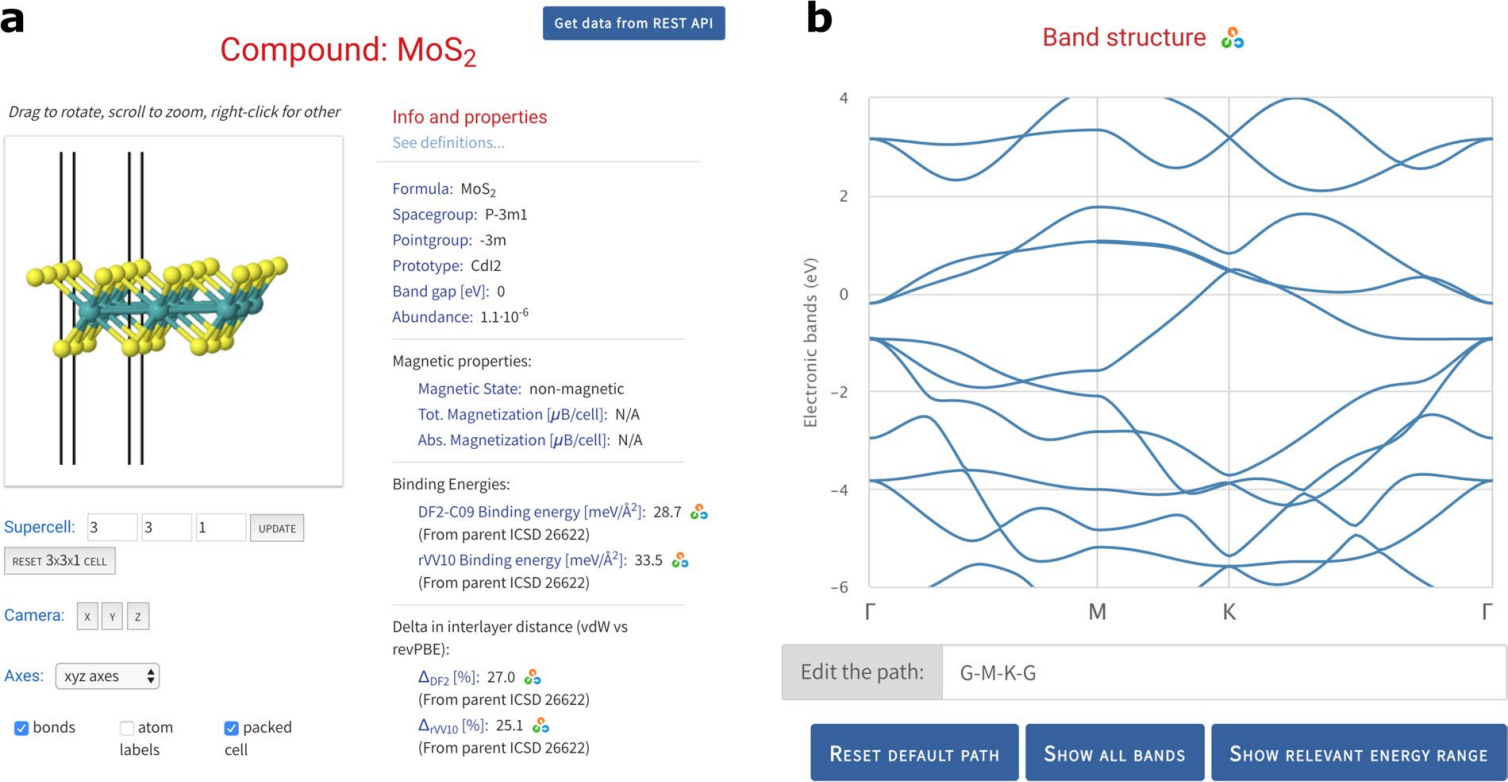
DISCOVER section on “*2D structures and layered materials*”^19^. The “ID card” of a material displays key computed properties, as well as interactive visualisations of the crystal structure **(a)**, the electronic band structure **(b)** and more. AiiDA icons link every piece of data to its corresponding node in the provenance graph that can be browsed through the EXPLORE interface shown in Fig. 4.

**Fig. 3:**
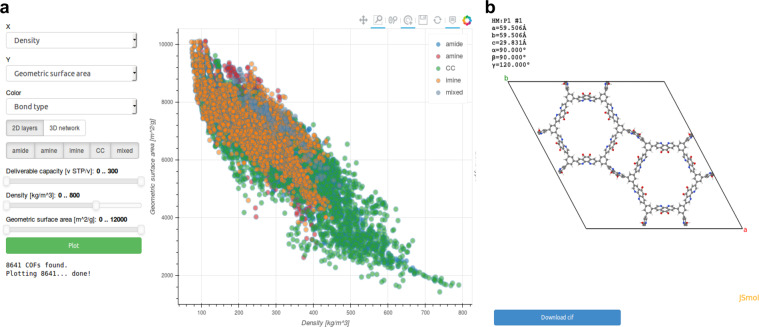
DISCOVER section on “*Covalent organic frameworks (COFs) for methane storage applications*”^21^, presenting almost 70000 COFs assembled *in silico*, together with their computed properties **(a)** and atomic structures **(b)** in the form of interactive figures that mirror those published in the corresponding peer-reviewed paper.

Researchers can leverage the full power of the approach by adding interactive DISCOVER and EXPLORE interfaces to their datasets in order to provide further layers of accessibility, interoperability and reproducibility (see also section Reproducibility below). DISCOVER sections focus on curated data, presented in the form of tailored interactive visualisations. For example, in the DISCOVER section “*2D structures and layered materials*”^19^, users can browse the curated dataset discussed in ref. ^20^. After selecting a material, key properties of the compound are displayed on a detail page (Fig. 2a,b), which includes interactive visualisations of quantities, such as the crystal structure, the electronic band structure, as well as phonon eigenvectors and band structures. Figure 3 shows screenshots from another DISCOVER section on “*Covalent organic frameworks (COFs) for methane storage applications*”^21^, containing interactive versions of the static figures published in ref. ^22^. The research data underlying all DISCOVER sections on Materials Cloud is published in corresponding ARCHIVE records and citable through DOIs.

What differentiates Materials Cloud DISCOVER sections from other approaches to presenting materials data is that each piece of data in a DISCOVER section can be linked to a node in the AiiDA provenance graph for full reproducibility, as we discuss in the following.

### Reproducibility: EXPLORE

While making data FAIR simplifies and accelerates the sharing of knowledge, it is equally important to ensure that the knowledge being shared is reliable. Computational materials science involves running computer programs on digital inputs and producing digital outputs. Yet, historically, only *some* input and output data have been shared in the computational materials science literature, often in narrative form, making it unnecessarily difficult for peers to reproduce reported results. While storing and sharing *all* data may not be technically feasible or financially sensible, researchers (and reviewers) today should demand that the data provided is sufficient to reproduce the reported results in their entirety.

This simple and seemingly self-evident demand can be tedious and time-consuming to meet in practice. Researchers leave out pieces of information for a variety of reasons: data may appear trivial, irrelevant or too complex to provide in accessible form. The challenge of providing access to this data is amplified in studies that involve large numbers of materials or workflows with many different steps. The need to simplify and automate this task has been one of the drivers for the development of the AiiDA framework^16,22^.

**Fig. 4:**
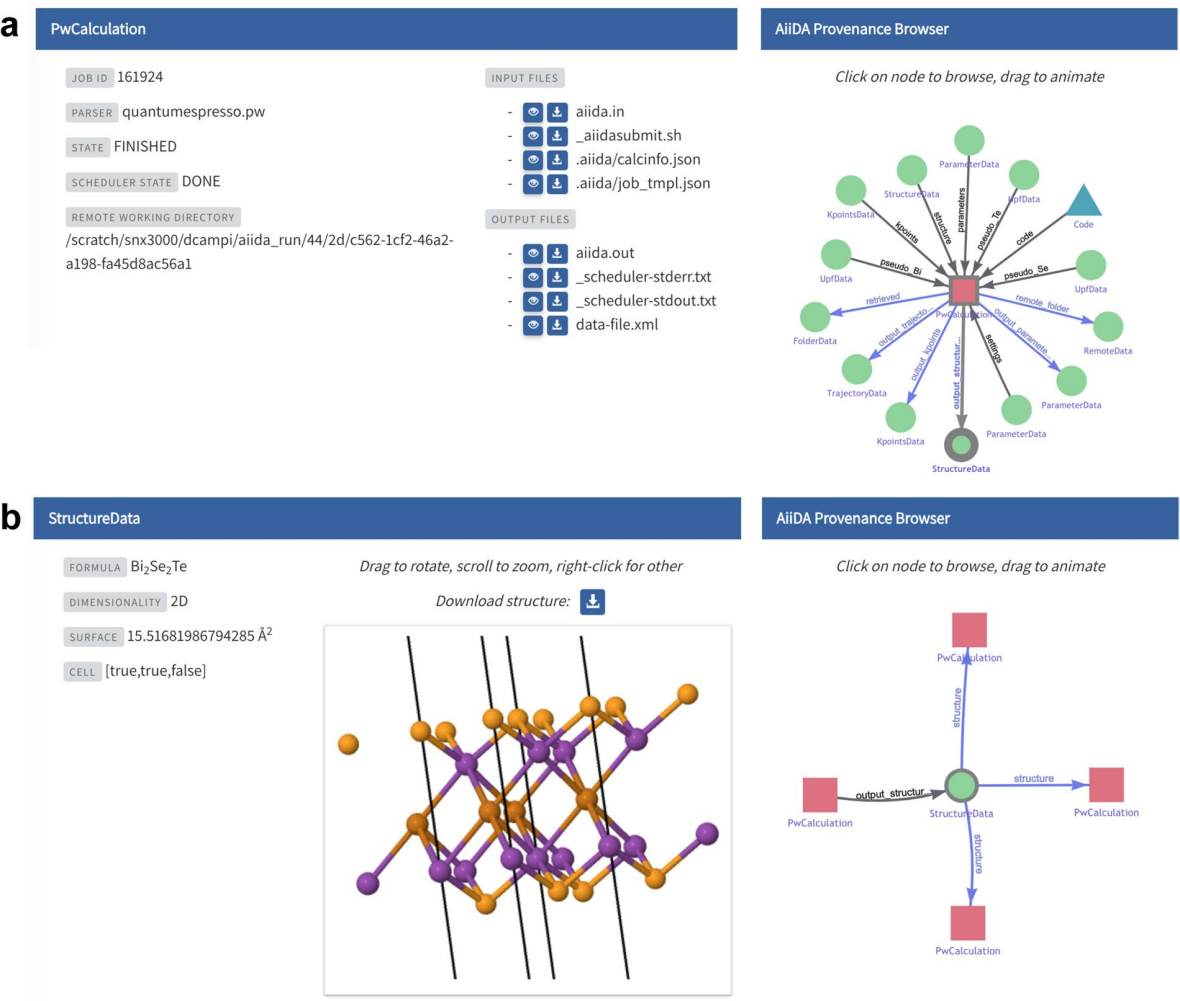
EXPLORE interface for AiiDA provenance graphs. **(a)** Interactive view of a calculation node (here representing a run of pw.x code from the Quantum ESPRESSO suite^27^), providing download links for all input and output files. The provenance browser on the right allows jumping to the visualisation of any input or output node of the calculation. **(b)** Interactive view of the atomic crystal structure returned by calculation **(a)**. The provenance browser indicates that this structure was used in three subsequent calculations. See the supporting information for the complete provenance graph.

AiiDA plays two main roles in the context of computational science: that of a manager of simulations, and that of a “stenographer” of events. The manager lets scientists interact seamlessly with remote high-performance computing (HPC) resources, and orchestrates computational workflows involving many steps, simulation codes, and possible paths. The stenographer records the data trail leading from the inputs to the results of a workflow, the *data provenance*, and stores it in databases tailored for efficient data mining of heterogeneous results. This includes information on who submitted the calculation, when the calculation was submitted, which computer and code were used, which inputs were used, which outputs were produced, as well as how these outputs are further used as inputs to the next calculation (see Fig. 4). Further details on the AiiDA provenance model can be found in the dedicated section of ref. ^15^.

Since long-term data storage is more expensive than the short-term storage used during simulations, it is often not reasonable to preserve all output data. Which output data to store is decided by the AiiDA plug-in for the code in question – for example, in a density-functional theory calculation, total energies, electronic band structures and log files might be stored by default, while Kohn-Sham wave functions might be discarded. The overarching principle, however, is that *all information needed to reproduce the outputs must be preserved*, even if not all intermediate files are persisted. By combining this information pertaining to individual calculations with the logical relationships between successive calculations, AiiDA provides reproducibility of entire workflows out of the box.

Scientists who use AiiDA for their calculations can choose to upload their AiiDA databases to the ARCHIVE in order to complement their published research with a comprehensive record of their calculations. When they do so, peers can browse the AiiDA graph in the corresponding EXPLORE section, as shown in Fig. 4: nodes of the graph represent either calculations or pieces of data. Each node is linked to its parent and child nodes, which can be traversed via the provenance browser. Dedicated visualisations make the content of nodes intuitively accessible, and allow downloading individual pieces of data (such as crystal structures or input files). Subject experts, on the other hand, can install AiiDA on their computer, import the AiiDA database with a single command, and continue their own research from where the authors of the original work left off.

In this context, AiiDA plays a role similar to Git (by tracking scientific workflows) while Materials Cloud plays a role similar to GitHub (a platform to share, browse and visualise all that has been tracked by AiiDA). Rather than trying to define and enforce a global schema and ingest all submissions into one monolithic database, Materials Cloud adopts the “repository of repositories” model of GitHub *et al*. and provides each submission with its own space. By using the AiiDA provenance model, Materials Cloud contributors nevertheless benefit from a unified user experience for browsing and searching for data and simulations. They can rely on standardised AiiDA data types, where appropriate, while AiiDA’s flexible plug-in system supports adding new types or extending existing ones to fit the specific purpose of the research undertaken. Finally, generic application programming interfaces, such as OPTIMADE (optimade.org), can operate across databases and provide features such as platform-wide crystal structure search.

### Simulation services: WORK and AiiDAlab

While DISCOVER, EXPLORE, and ARCHIVE enable the dissemination of results that have already been computed, in the WORK section users can launch well-defined data generation and analytics workflows directly from the web browser, thus making these workflows accessible to a wider user base of students, experimental scientists, and computational scientists.

**Fig. 5:**
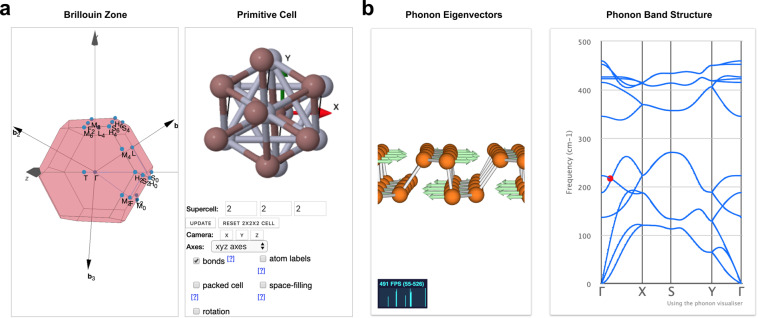
Tools in the WORK section. **(a)** SeeK-path tool^44^ for finding and visualising paths in reciprocal space, here showing the Brillouin zone of InHg. **(b)** Interactive visualiser for lattice vibrations (adapted from henriquemiranda.github.io/phononwebsite), here of two-dimensional phosphorene. Shown is the phonon eigenvector (left) corresponding to the red dot in the phonon band structure (right)^44^.

On the one hand, this includes stand-alone tools that run computationally inexpensive simulations and produce immediate results: ranging from tools that help plotting electronic band structures (Fig. 5a) or visualising lattice vibrations (Fig. 5b) to tools leveraging machine learning methods to predict materials properties. The underlying docker technology (docker.com) makes it possible to support a diverse set of software frameworks on the same platform, allowing for custom solutions that are adapted to the specific tool in question.

**Fig. 6:**
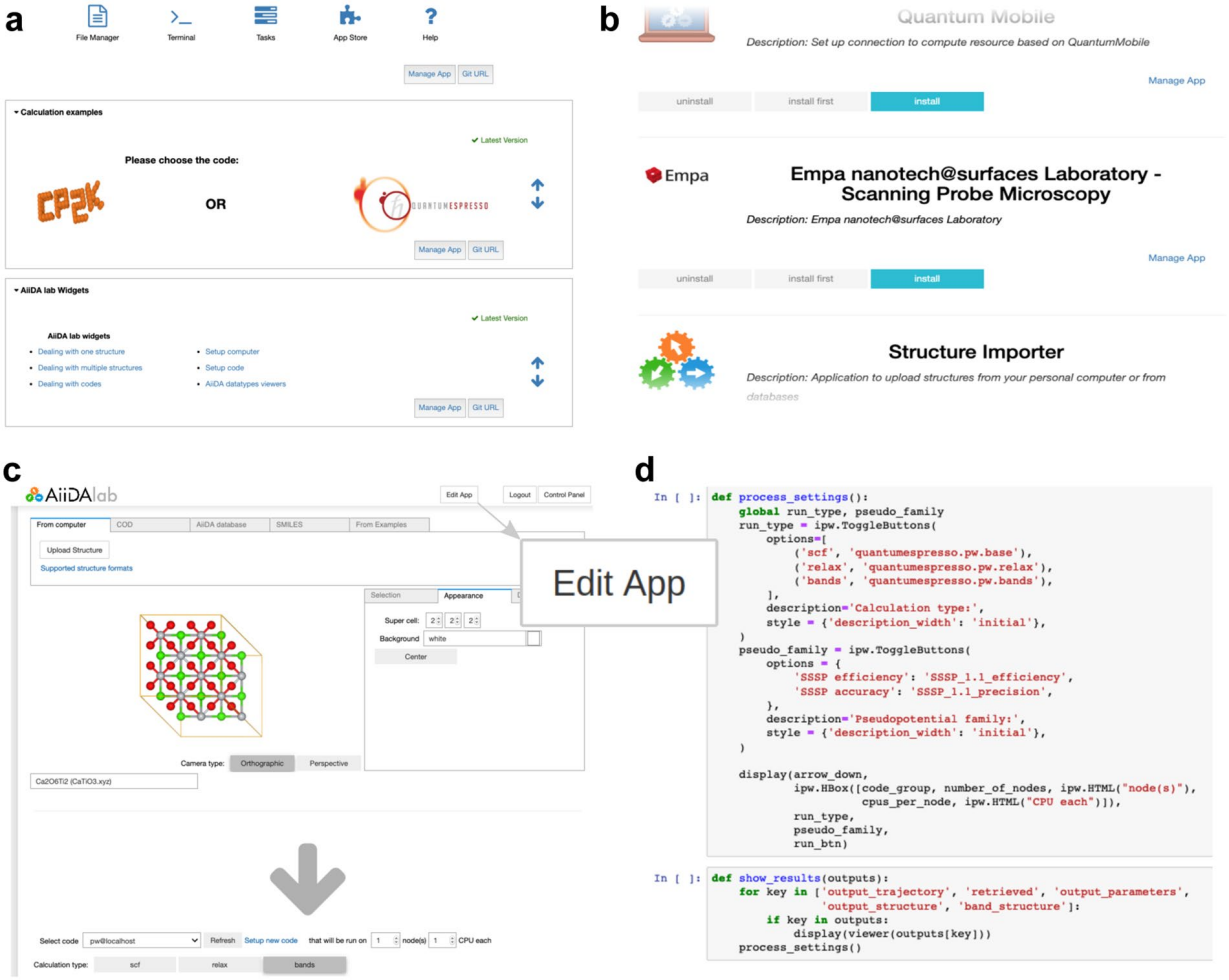
AiiDAlab simulation environment. **(a)** Landing page with an overview of the applications installed. **(b)** “App store” for managing applications. **(c)** Application that computes the optimised crystal structure of an input material as well as its electronic band structure along standardised paths. Clicking “Edit App” switches to the source code editor **(d)** of the underlying Jupyter notebook.

On the other hand, the WORK section focuses on the AiiDAlab, an ecosystem for applications powered by the AiiDA workflow manager (materialscloud.org/aiidalab). AiiDAlab aims to remove barriers related to the set up and installation of simulation software by providing access to applications for launching and controlling computational workflows directly from the web browser. Users log in to a private, containerised environment that provides a persistent work space for storing apps, their AiiDA database, and file repository (Fig. 6a,b). AiiDAlab apps let users connect their own computational resources anywhere in the world in order to run their workflows of interest at the scale they desire. Users can upload data to the platform either directly from their computer, or from connected open databases such as the Crystallography Open Database^23^ or any database implementing the OPTIMADE standard (optimade.org), including AFLOW (aflow.org^8^), COD (crystallography.net/cod^23^), TCOD (crystallography.net/tcod^24^), MPDS (mpds.io^25^), Materials Project (materialsproject.org^9^), NOMAD (nomad-coe.eu^12^), Open Materials Database (openmaterialsdb.se), OQMD (oqmd.org^10^), and Materials Cloud itself.

The intuitive graphical interface makes AiiDAlab applications a great vehicle for sharing turnkey solutions with non-specialists, be it computational scientists from another discipline or experimental researchers with no programming experience.

From a technological perspective, AiiDAlab applications are Jupyter notebooks (jupyter.org) containing instructions for the AiiDA workflow manager, which are transformed into an interactive web application (see Fig. 6c,d). This design has two important implications for app *development*: First, the widespread adoption of Python and Jupyter notebooks in data science in general^26^ and computational materials science in particular makes most researchers in the field potential app developers. In particular, thanks to Jupyter widgets, interactive web interfaces can be written in a few lines of Python, and no longer require knowledge of JavaScript. And second, AppMode lets developers switch between the graphical app layout (Fig. 6c) and the Python development environment (Fig. 6d) at the click of a button. Apps can be edited live in the browser, and developers have the full power of the Python programming language at their fingertips.

AiiDAlab encourages the sharing of workflows and visualisations via an App store model: in a first step, developers register their application on the application registry (aiidalab.github.io/aiidalab-registry). Once registered, other users can then install the app via the built-in application manager (Fig. 6b) and access it from their home screen (Fig. 6a). The source codes of the AiiDAlab, AppMode, and AiiDA itself are released under the permissive MIT open-source license (see code availability statement), enabling re-deployment of the AiiDAlab platform both in academic and in corporate environments. When a local, self-contained AiiDAlab environment is desired – e.g., for educational purposes – users can download the Quantum Mobile virtual machine that runs on Linux, MacOS, and Windows (see section Education and outreach).

### Education and outreach: LEARN and Quantum Mobile

**Fig. 7:**
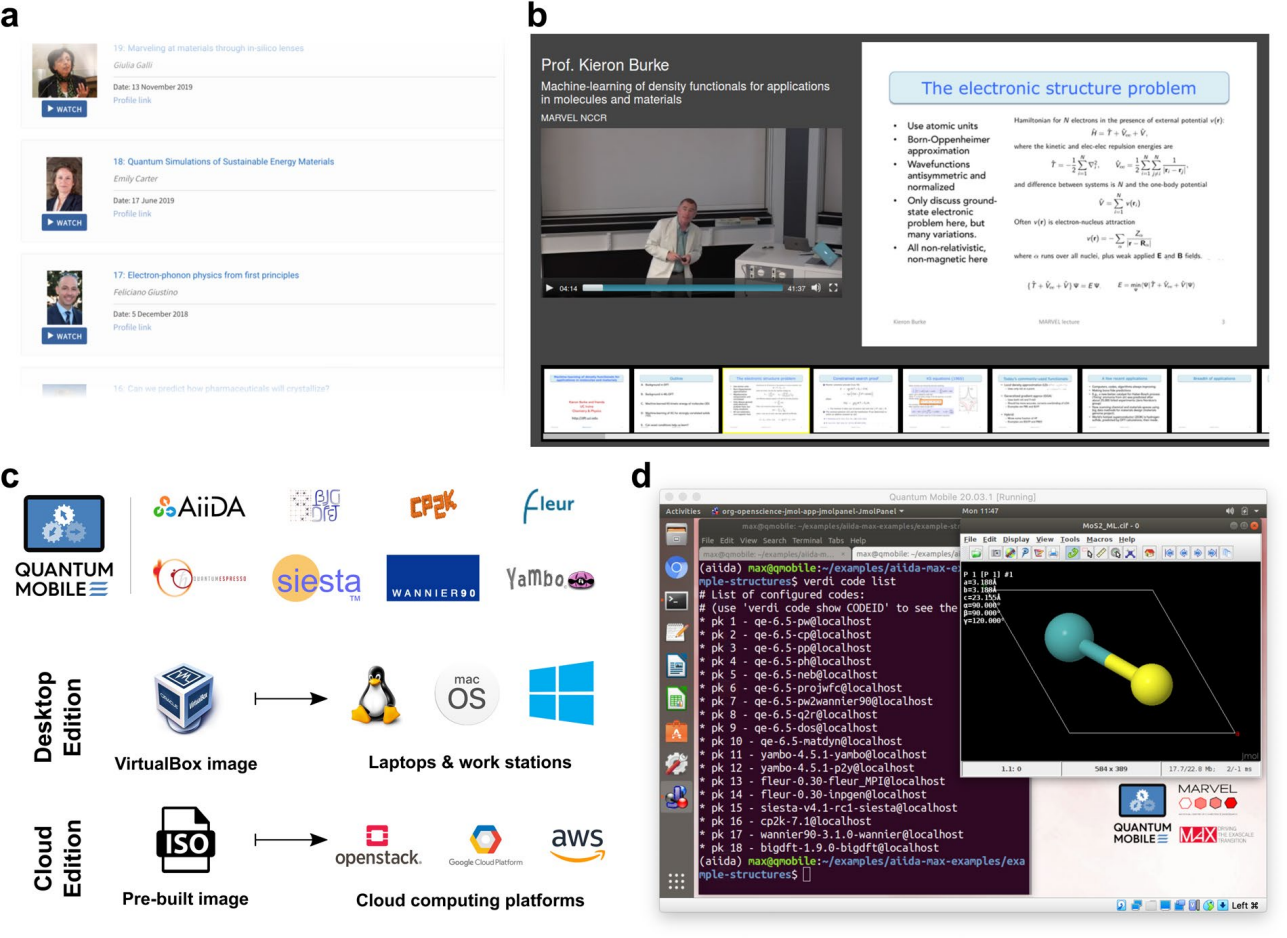
Education and outreach. (**a**) MARVEL distinguished lectures available in the LEARN section. (**b**) Slideshot player with slide synchronisation and slide-based browsing. (**c**) Simulation codes provided with the Quantum Mobile virtual machine, and deployment schemes for the Desktop and Cloud Edition. (**d**) Screenshot of the Quantum Mobile desktop.

The LEARN section of Materials Cloud hosts video lectures, tutorials, and seminars in computational materials science (Fig. 7a). Lectures in collaboration with CECAM (cecam.org) include the “Classics on Molecular and Materials Simulations”, dedicated to record pioneering contributions in the field, and the “Mary Ann Mansigh conversations” in which outstanding representatives from computational science share their perspective on how modelling affects society. Videos are grouped by topic or event, presented together with accompanying materials, and slides where available. The Slideshot video player shows video and slides side by side, and keeps them in sync (Fig. 7b, slideshot.epfl.ch).

Besides the educational materials in the LEARN section, students can also download the Quantum Mobile virtual machine for computational materials science (Fig. 7c) from the WORK section. Quantum Mobile is based on Ubuntu Linux and comes pre-installed with a collection of open-source software packages for quantum-mechanical calculations including Quantum ESPRESSO^27^, Yambo^28^, fleur^29^, Siesta^30^, CP2K^31^, and Wannier90^32^.

Furthermore, it includes the Standard Solid State Pseudopotential Library (SSSP)^33,34^, various visualisation tools (jmol^35^, XCrySDen^36^, gnuplot, grace), a job scheduler (Slurm) and a build environment with C, C++ and Fortran compilers as well as scientific and MPI libraries. AiiDA and the AiiDAlab environment are pre-configured, including AiiDA plug-ins for each of the ab initio codes listed above, ready to be used out-of-the-box (Fig. 7c,d).

Quantum Mobile provides a uniform environment for quantum mechanical materials simulations and runs on most popular operating systems, including Linux, MacOS and Windows, via the VirtualBox software (virtualbox.org). Contrary to other encapsulation strategies, such as Docker, students interact with a familiar graphical desktop, shown in Fig. 7d. Since its first release in November 2017, Quantum Mobile has been updated continuously and its Desktop and Cloud Editions have been used in lecture courses at EPFL, ETHZ, and Ghent University (compmatphys.org) as well as in numerous tutorials on electronic structure methods, molecular simulations, and AiiDA (see materialscloud.org/quantum-mobile), where it helps reducing the time needed for installation and configuration of software.

The modular design of Quantum Mobile takes into account that one size does not fit all: its components (simulation codes, tools, data) are encapsulated in reusable, individually tested components (see Code availability statement). Teachers can pick and choose from a growing repository of more than 30 components and build their own version of Quantum Mobile containing just the tools they need.

## Discussion and Outlook

The increasing availability and standardisation of infrastructure-as-a-service (IaaS) make it possible to share the findings and capabilities developed by computational materials scientists not only with peers who possess journal subscriptions and specialist software, but with anyone who is familiar with using a web browser. Materials Cloud aims to provide a comprehensive platform for open science that (i) can be accessed free of charge by anyone and (ii) allows anyone to submit raw and curated data, with light moderation for adherence to scientific scope and basic data interoperability and reusability standards (ARCHIVE and DISCOVER sections). Data records are findable through DOIs, with metadata indexed in relevant search engines, and data persistence guaranteed for 10 years from the time of submission. When complemented by the AiiDA computational infrastructure, Materials Cloud stores and exposes the provenance of computed results in meticulous detail – including data and metadata of all intermediate steps leading up to them – in order to ensure reproducibility, adding further layers of interoperability and reusability to its implementation of the FAIR principles^6^ (EXPLORE section). Finally, the WORK section offers online computational tools as a service, both in stand-alone form and on the AiiDAlab platform for sharing turnkey solutions.

Historically, the project started thanks to the support of the Swiss National Science Foundation, through the National Centre for Computational Design and Discovery of Novel Materials (NCCR MARVEL, started in 2014 and slated to run until 2026), and of the European Commission, through the Centre of Excellence for Materials Design at the Exascale (MaX, from 2015 onwards). In the years since its launch, further partners have joined (materialscloud.org/home#partners), and the Materials Cloud has grown steadily as a repository for sharing research data, workflows, and tools well beyond its original communities. At the same time it provides a laboratory for exploring ideas and models for future digital infrastructures – for example, automated turnkey simulations encoded in the AiiDAlab that can be re-deployed publicly or internally by academic institutes, research centres, and companies through a clone of the platform on their own hardware.

While the content on Materials Cloud is easily *accessed* through the web browser, *submitting* new tools and interactive visualisations still requires technical expertise. The consortium is working on lowering this barrier and on reducing the associated workload of platform administrators by moving in the direction of a platform-as-a-service architecture. Finally, the governance model of Materials Cloud will evolve, driven by the recently established Materials Cloud GO FAIR Implementation Network (go-fair.org/implementation-networks/overview/materials-cloud/) and by the computational materials science community at large.

One major challenge in the field is that of finding a common language for information exchange between OSPs. Notably, the computational materials science community started in 2016 to develop an API standard for exchanging materials data stored in repositories worldwide: the OPTIMADE consortium (optimade.org) meets yearly to update and extend its specification, with involvement of all major players and welcoming any new arrivals. Other important efforts range from the collection of semantic assets^37^, over the design of new ontologies^38^, to specifications of interoperable data formats^39,40^. Once these efforts converge, they can be connected to existing infrastructures for structured web data (schema.org) – until that point, automated provenance tracking can help make data more future-proof by casting a wide net for metadata that enables compliance with different metadata standards *a posteriori*^41^.

Another important question is which models are best suited to secure the continued development and maintenance of digital research infrastructures like Materials Cloud in the very long term, beyond the horizon of any project-based research funding streams. Analogies can be drawn to other established research infrastructures – ranging from particle accelerators over radio telescopes to libraries – where key services are provided to the scientific community, funded either by the public or in the form of public-private partnerships. Given the unprecedented availability of computational power (top500.org), the pervasiveness of computational (materials) science in the scientific literature^42^, and its relevance to pressing societal challenges^43^, maintaining functional digital research infrastructures would seem like a forward-looking investment. Not only is the required funding minimal compared to some of the other efforts mentioned above, but the cost of digital infrastructure also increases only marginally with the number of users served – a sharp contrast to the finite capacity of brick-and-mortar facilities. In conclusion, it is worth recalling that a considerable share of our personal and professional life has already gone digital, with early pioneering enterprises capturing large shares of the new emerging markets. The implications for science, and the urgent need for digital research infrastructures seem obvious.

## Methods

**Fig. 8:**
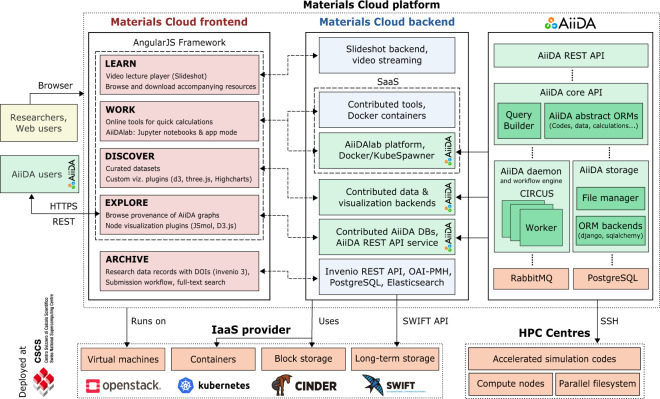
Materials Cloud architecture diagram. Independent frontends for LEARN, WORK, DISCOVER, and EXPLORE based on AngularJS are powered by different backends, including AiiDA’s REST API, tools encapsulated in docker containers and a JupyterHub running one docker container per AiiDA user.

Materials Cloud’s modular architecture, sketched in Fig. 8, is designed to enable updates of individual sections without affecting the rest of the service. The top-level web user interface is presented through a set of AngularJS applications with one app per section. Section content is served either directly by the corresponding application (LEARN, DISCOVER, EXPLORE) or through user interfaces provided by containerised content (WORK, DISCOVER). The overall Materials Cloud theme is based on the Bootstrap (getbootstrap.com) and Material Design (material.angularjs.org) frameworkss; and individual sections use a range of visualisation libraries, including Highstock/Highcharts (highcharts.com), D3js (d3js.org), JSMol (jsmol.sourceforge.net/), and Vis (visjs.org).

A slideshot server provides the API to serve videos and slides to the LEARN section. Tools in the WORK section are encapsulated in docker containers and define their own web frontends. The AiiDAlab is a customised JupyterHub that is isolated from the rest of the platform and runs on a separate server. Every AiiDAlab account is associated with a private container, including persistent storage and compute resources, and can be connected to high-performance computing resources owned by the account holder. Containerised contributions to WORK and DISCOVER may use different Python-based frameworks, such as Flask (flask.palletsprojects.com), or Bokeh (bokeh.org).

In the EXPLORE section, the frontend JavaScript application talks directly to the standardised AiiDA application programming interface (API). This representational state transfer (REST) API provides access to calculations, workflows, codes, and data stored in the AiiDA graph, and makes them available in the JavaScript Object Notation (JSON) format. The AiiDA REST API ships together with AiiDA; besides serving static AiiDA databases on the Materials Cloud, AiiDA users can take advantage of the same JavaScript application powering EXPLORE to browse their own local AiiDA database. For more details, see Fig. S1 in the supplementary materials.

The ARCHIVE section is only loosely coupled to the platform. Files associated with ARCHIVE records are stored in an OpenStack Swift Object Store and backed up daily to tape (user.cscs.ch/storage/object_storage). The ARCHIVE server hosts the database containing the metadata associated with records, and delegates requests for associated files to the object store via short-lived unique URLs. The implementation is based on the highly scalable Invenio 3 framework (invenio-software.org), which is developed at CERN and also powers the Zenodo repository.

Materials Cloud is operated by the Materials Cloud team (materialscloud.org/team), a group of developers at EPFL, and deployed on virtual machines running in an OpenStack cloud computing platform (openstack.org) at the Swiss National Supercomputing Centre (CSCS). All production servers are duplicated, following standard web development practises (see Fig. S2 in the supplementary materials for details). In order to prevent loss of log files and user data, backups are taken periodically and stored in the object storage service at CSCS. A server at a different physical location monitors availability and basic functionality of all production services every 60 seconds and notifies maintainers in case of unexpected deteriorations of service.

Deployment is automated using Ansible playbooks (ansible.com), which allow software provisioning, configuration management, and application deployment on remote machines over SSH. The use of automated Ansible roles, together with Materials Cloud’s modular architecture and the widely available OpenStack infrastructure, simplifies the redeployment of Materials Cloud (or components of it) in other locations, e.g., for the purpose of load balancing, federation of service, or in-house use.

## Supplementary information

Supplementary Information

## Data Availability

The datasets discussed in this paper^19,21^, as well as all datasets underlying the Materials Cloud DISCOVER and EXPLORE sections are available on the Materials Cloud Archive (archive.materialscloud.org) under Creative Commons licenses.

## Code availability

The source code of AiiDA, the AiiDAlab, Appmode (github.com/oschuett/appmode), and most AiiDAlab applications is released under the MIT open-source license, and made available under the aiidateam (github.com/aiidateam) and aiidalab (github.com/aiidalab) GitHub organisations.

The Quantum Mobile virtual machine can be downloaded from materialscloud.org/quantum-mobile. Its source code (in the form of ansible roles and playbooks) is released under the MIT license and made available under the marvel-nccr GitHub organisation (github.com/marvel-nccr).
