# Supplementary material for: Materials Cloud, a platform for open computational science
Source: Sci Data. 2020 Sep 8;7:299. doi: 10.1038/s41597-020-00637-5 (PMC7479138; doi:10.1038/s41597-020-00637-5)
Supplement: Supplementary file 1 — Supplementary Information [file 41597_2020_637_MOESM1_ESM.pdf]

Supporting Information for: Materials Cloud, a platform for open computational science

Leopold Talirz[1,2,3,†,*], Snehal Kumbhar[1,2,†], Elsa Passaro[1,2,3,†], Aliaksandr V. Yakutovich[1,2,3], Valeria Granata[1,2], Fernando Gargiulo[1,2], Marco Borelli[1,2], Martin Uhrin[1,2], Sebastiaan P. Huber[1,2], Spyros Zoupanos[1,2], Carl S. Adorf[1,2], Casper W. Andersen[1,2], Ole Schütt[1,4], Carlo A. Pignedoli[1,4], Daniele Passerone[1,4], Joost VandeVondele[1,5,6], Thomas C. Schulthess[1,5,6], Berend Smit[1,3], Giovanni Pizzi[1,2,*], and Nicola Marzari[1,2,*]

[1]National Centre for Computational Design and Discovery of Novel Materials (MARVEL), École Polytechnique Fédérale de Lausanne, CH-1015 Lausanne, Switzerland
[2]Theory and Simulation of Materials (THEOS), Faculté des Sciences et Techniques de l'Ingénieur, École Polytechnique Fédérale de Lausanne, CH-1015 Lausanne, Switzerland
[3]Laboratory of Molecular Simulation (LSMO), Institut des Sciences et Ingenierie Chimiques, Valais, École Polytechnique Fédérale de Lausanne, CH-1951 Sion, Switzerland
[4]nanotech@surfaces laboratory, Swiss Federal Laboratories for Materials Science and Technology (Empa), CH-8600 Dübendorf, Switzerland
[5]Swiss National Supercomputing Centre (CSCS), CH-6900 Lugano, Switzerland
[6]ETH Zürich, Switzerland
[†]These authors contributed equally to this work.
[*]Corresponding authors: Leopold Talirz (leopold.talirz@gmail.com), Giovanni Pizzi (giovanni.pizzi@epfl.ch), Nicola Marzari (nicola.marzari@epfl.ch)

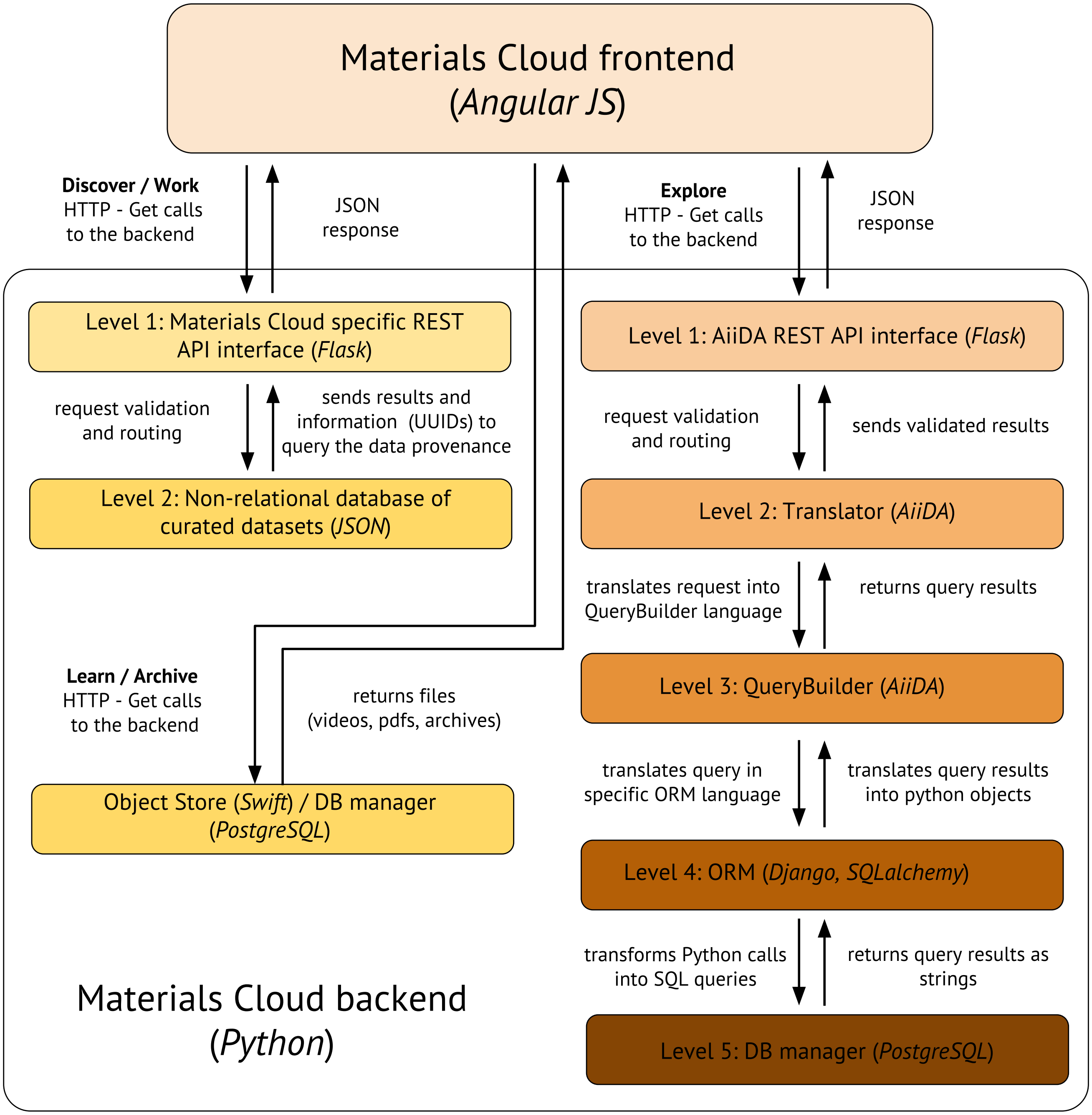

Figure S1. Data flow between Materials Cloud frontend and backend. Left: Data flow for ARCHIVE, DISCOVER and WORK. Right: AiiDA REST API, flow starting from the browser request (top) via data validation, parsing and translation into the AiiDA query language down to the database query (bottom). Response data then follows the reverse path and is returned to the browser in JSON format.

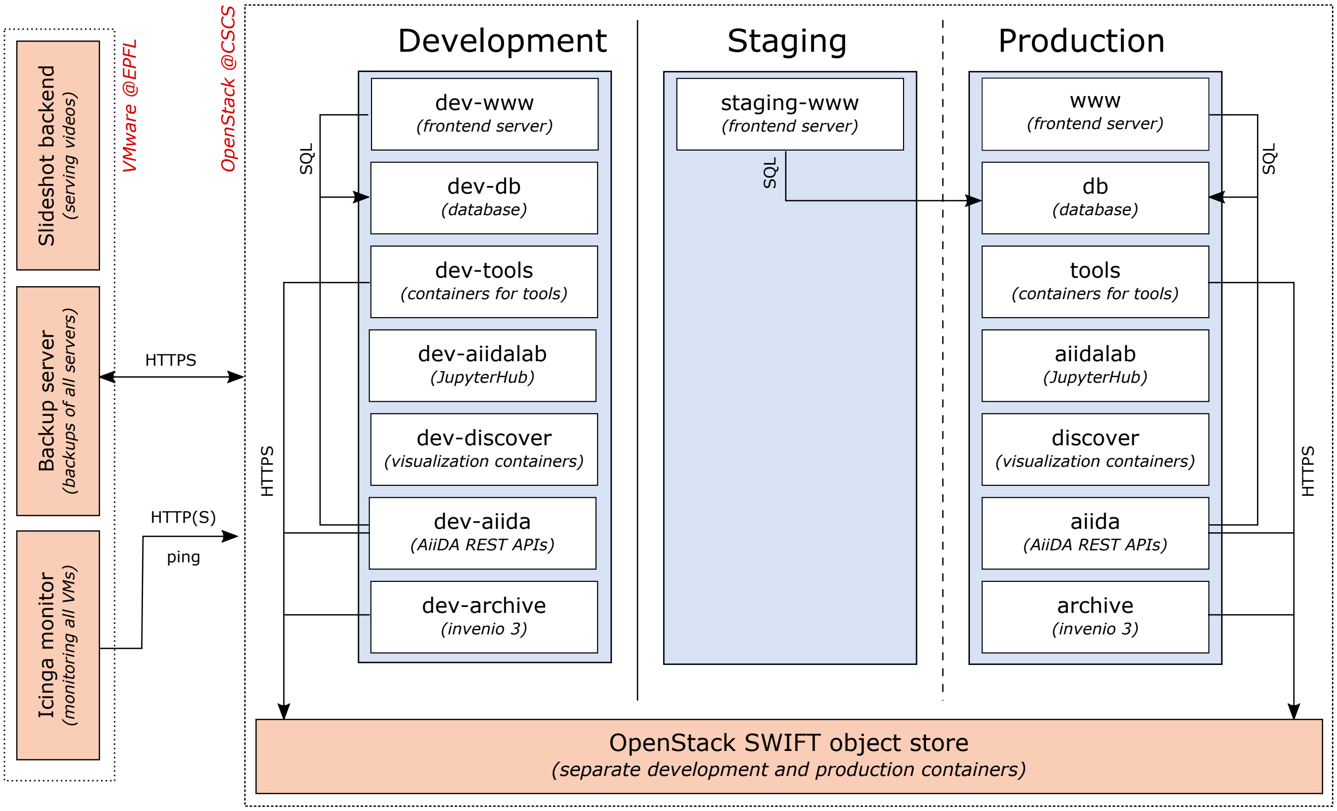

Figure S2. Materials Cloud deployment diagram. Services are split across multiple virtual machines, each with at least one clone for development. The frontend servers communicate with all other servers via HTTPS (not shown).

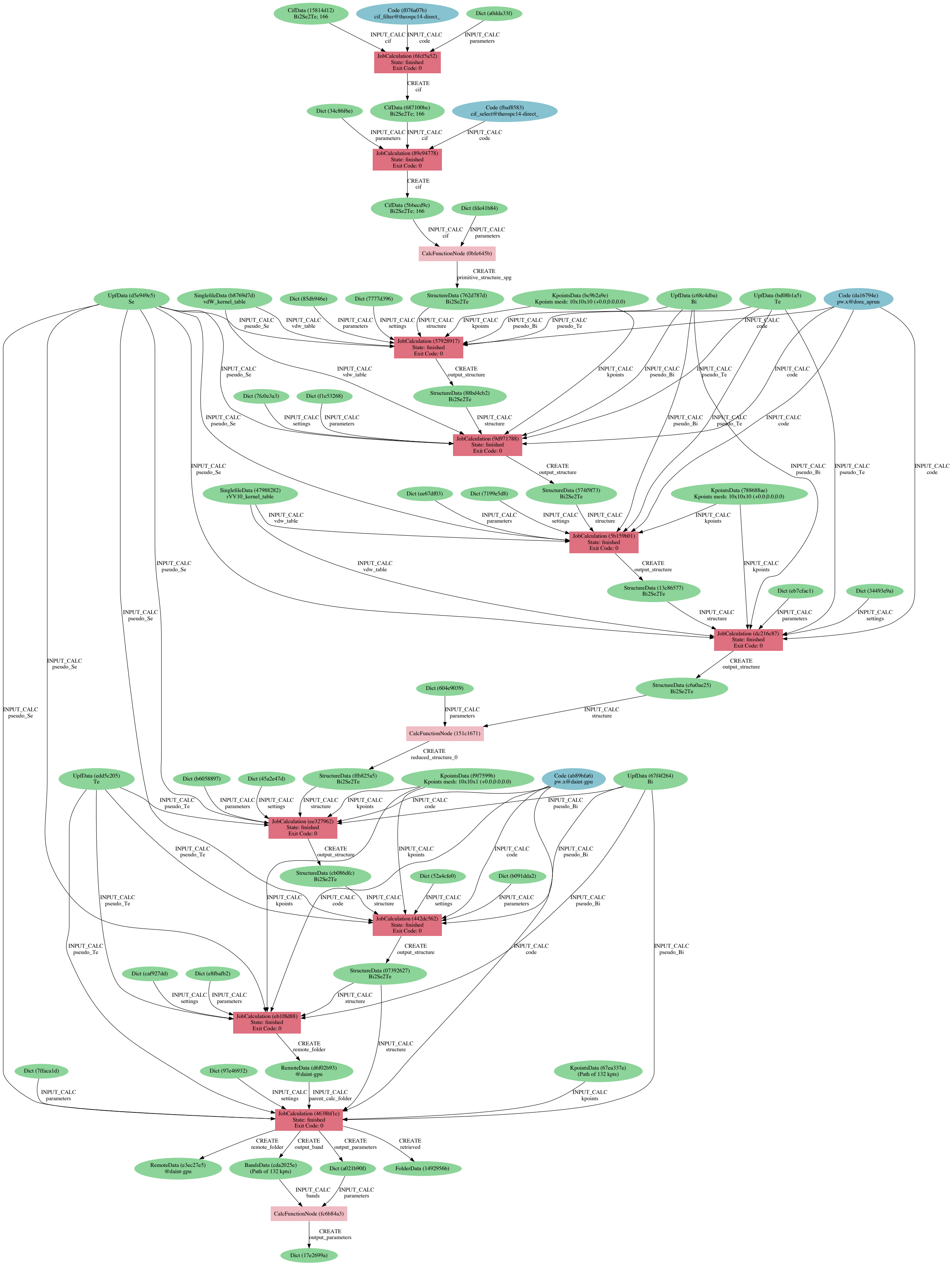

Figure S3. Complete AiiDA provenance graph for the electronic band structure calculation of Bi_2Se_2Te, as generated by `verdi node graph generate 4638bfc1` on the AiiDA database from reference[1].

References

1. Mounet, N. *et al.* Two-dimensional materials from high-throughput computational exfoliation of experimentally known compounds. *Mater. Cloud Arch.* 10.24435/materialscloud:2017.0008/v3 (2019).
